# Occurrence of respiratory viruses among outpatients with diarrhea in Beijing, China, 2019–2020

**DOI:** 10.3389/fmicb.2022.1073980

**Published:** 2023-01-12

**Authors:** Lingyu Shen, Hanqiu Yan, Weihong Li, Yi Tian, Changying Lin, Baiwei Liu, Yu Wang, Lei Jia, Daitao Zhang, Peng Yang, Quanyi Wang, Zhiyong Gao

**Affiliations:** ^1^Institute for Infectious Diseases and Endemic Diseases Prevention and Control, Beijing Center for Disease Prevention and Control, Beijing, China; ^2^Institute of Pathogen Biology, Chinese Academy of Medical Sciences and Peking Union Medical College, Beijing, China

**Keywords:** diarrhea, respiratory viruses, SARS-CoV-2, genotype, clinical characteristics

## Abstract

**Objectives:**

To investigate respiratory virus infections in diarrhea cases and identify the risk of respiratory virus transmission through feces.

**Methods:**

Fecal specimens were collected from diarrhea cases in enteric disease clinics in Beijing, China, from 2019 to 2020. Cases that tested negative for norovirus, rotavirus, sapovirus, astrovirus, and enteric adenovirus were included in the study. Real-time RT-PCR was used to detect 16 groups of respiratory viruses, and the major viruses were genotyped. Viruses isolation and digestion of clinical specimens and nucleic acid by artificial gastric acid or artificial bile/pancreatic juice were used to evaluate the risk of respiratory virus transmission through feces.

**Results:**

A total of 558 specimens were collected and 47 (8.42%) specimens were detected positive, 40 (13.33%, 40/300) in 2019, and 7 (2.71%, 7/258) in 2020, including 20 (3.58%) for human rhinovirus (HRV), 13 (2.32%) for Bocavirus (BoV), 6 (1.08%) for parainfluenza virus I (PIV), 4 (0.72%) for coronavirus (CoV) OC43, 3 (0.54%) for respiratory syncytial virus (RSV) A, and 1 (0.18%) for both BoV and CoV OC43. Syndrome coronavirus 2 (SARS-CoV-2) and other viruses were not detected in this study. Eight genotypes were identified in the 13 HRV specimens. BoVs 1 and 2 were identified in nine BoV specimens. HRV infectious virions were successfully isolated from 2 clinical specimens and clinical specimens of HRV, RSV, PIV, and CoV could not be detected after 4 h of digestion and their nucleic acid could not be detected after 2 h of digestion by artificial gastric acid or artificial bile/pancreatic juice.

**Conclusion:**

There may be a risk of respiratory virus transmission from diarrhea cases, and interventions against SARS-COV-2 epidemics are also effective for other respiratory viruses.

## Introduction

Diarrhea is the main symptom caused by enteric pathogen infection; however, several respiratory virus infections also present with this symptom. Severe acute respiratory syndrome coronavirus 2 (SARS-CoV-2), which emerged in the final months of 2019, causes severe acute respiratory diseases, multiple organ injuries, and fatal outcomes (Huang et al., 2020). SARS-CoV-2 enters cells via the angiotensin-converting enzyme 2 (ACE2) receptor, which is highly expressed in esophageal epithelial cells and absorptive enterocytes from the ileum and colon, suggesting possible fecal transmission (Hoffmann et al., 2020; Huang et al., 2020). An incidence of gastrointestinal symptoms of COVID-19 showed that diarrhea and vomiting were the most common gastrointestinal symptom in children and adults, from 2.0 to 49.5% and 3.6 to 66.7%, respectively (Tian et al., 2020). Coronavirus (CoV) OC43 labeled “gastric flu” is also transmitted via the respiratory tract, and 57% of infected cases have gastrointestinal symptoms (Freymuth et al., 1997; Vijgen et al., 2005; Openshaw, 2009).

A prospective observational study found that among diarrhea cases, the RNA of human influenza A virus (IAV) and human influenza B virus (IBV) became undetectable in sputum on days 7 and 10 after infection, respectively, although it could be detected in feces 24 days after infection. In addition, their RNA and antigens were detected in intestinal tissues, and diarrhea symptoms were observed in patients whose feces tested positive (Hirose et al., 2017; Karch et al., 2021). These findings suggest that the human intestine may be an additional target organ for IAV/IBV infections.

Bocavirus (BoV) was first identified in Swedish children with acute respiratory infections (ARTIs) in 2005 and divided into four genotypes (BoVs 1 to 4) (Koskenvuo et al., 2008; Kawai et al., 2012). After the introduction of rotavirus A (RVA) vaccines by the National Immunization Programs (NIP) worldwide, BoV is currently considered an emerging virus that could be associated with cases of acute gastroenteritis (AGE), and all these genotypes have been detected in fecal samples (Koskenvuo et al., 2008; Khamrin et al., 2012). Both respiratory syncytial virus (RSV) and human metapneumovirus (MPV) are the main pathogens causing lower respiratory tract infections in children, and approximately 10% of positive cases had vomiting and 3.5% had diarrhea in the previous studies (Garten et al., 2009; Al-Mahrezi et al., 2012).

Human rhinovirus (HRV) has been associated with lower respiratory tract (LRT) illness and more severe clinical outcomes in pediatric and other vulnerable populations. At present it has the most well-defined role in causing the “common cold” (Hayden, 2004; Kiang et al., 2007). HRV is the closest genetic relative of the enterovirus (EV) transmitted through feces, and similar to some EVs, they use intercellular cell adhesion molecule-1 (ICAM-1) as their primary cellular receptor (Xiao et al., 2001; Lewis et al., 2018).

Several respiratory viruses, including CoV, IAV, IBV, BoV, RSV, MPV, and HRV, can be transmitted by the respiratory tract and cause diarrhea or have the same cellular receptor as the gut virus. Therefore, this study aimed to investigate the epidemiological distribution and clinical characteristics of respiratory viruses among outpatients with diarrhea in Beijing to further understand the causes of diarrheal diseases and provide information on the risk of respiratory virus transmission through feces.

## Materials and methods

### Patients

Three enteric disease clinics located in the Chaoyang district of the urban area, Tongzhou district of the suburbs, and Huairou district of the outer suburbs were enrolled in this study. Diarrhea cases were defined as patients with three or more loose fecal samples within a 24 h period. Fecal samples of children aged 0–10 years with diarrhea were collected in clinics in Chaoyang district and Tongzhou district, and samples of the whole age group were collected in Huairou district. Thereafter, 10–15 samples per month were collected from each district. These cases were first detected for norovirus, rotavirus, sapovirus, astrovirus, and enteric adenovirus, and negative cases were included in the study.

### Real-time PCR for respiratory viruses

Viral RNA was extracted from 140 μL of a 10% (w/v) fecal suspension in phosphate-buffered saline using the QIAamp Viral RNA Mini Kit (QIAGEN Co., Ltd., Hilden, Germany). Approximately 60 μl of the total nucleic acid eluate was recovered in nuclease-free tubes. RNA was stored at −20°C until further use.

A panel of respiratory viruses, including influenza virus A (H1N1 pandemic 2009, H3N2) and B, RSV, parainfluenza virus I to IV (PIV I to IV), HRV, MPV, coronavirus (CoV NL63, OC43, 229E, and HKU1), and BoV, was detected in these specimens using commercial real-time RT-PCR kits (Jiangsu Uninovo Biological Technology Co., Ltd., China). Commercial real-time PCR kits (Applied Biological Technologies Co., Ltd., Beijing, China) was used to detect severe acute respiratory SARS-CoV-2.

Among specimens positive for respiratory viruses, a panel of diarrhea bacteria, and parasites, including *Escherichia* (EPEC, EAEC, ETEC, EHEC, and EIEC), *Vibrio parahaemolyticus, Yersinia enterocolitica, Bacillus atrophaeus, Aeromonas hydrophila, Campylobacter jejuni, Plesiomonas shigelloides, Aeromonas hydrophila, Campylobacter jejuni, Campylobacter coil, Clostridium difficile, Vibrio cholera, Vibrio vulnificus, Blastocystis hominis, Dientamoeba fragilis, Entameba histolytica, Giardia lamblia*, and *Cryptosporidium*, were detected using the commercial TaqMan™ Microbial Array Specialty Card (Thermo Fisher Scientific Co., Ltd., MA, USA). *Escherichia* (EPEC, EAEC, ETEC, EHEC, and EIEC) were also identified by the real-time PCR method (Qu et al., 2016) (Applied Biological Technologies Co., Ltd., Beijing, China).

### Phylogenetic analysis and genotyping of HRVs and BoVs

Fragments of the HRV VP4/2 region were amplified by nested PCR using a QIAGEN One-Step RT-PCR Kit (Wisdom et al., 2009). The first-round RT-PCR for 688 bp of HRV VP4/2 region was conducted by QIAGEN One-Step RT-PCR Kit (QIAGEN, Hilden, Germany) in a 50 μL reaction volume and performed using the following thermal cycling parameters: 50°C for 30 min; 95°C for 15 min; 40 cycles of 94°C for 30 s, 54°C for 1 min, and 72°C for 1 min; and a final extension at 72°C for 7 min. The second-round PCR for 563 bp of HRV VP4/2 region was conducted by AmpliTaq Gold™ 360 Master Mix (Thermo Fisher Scientific Co., Ltd., Massachusetts, USA) in a 50 μL reaction volume and performed using the following thermal cycling parameters: 94°C for 2 min; 25 cycles of 94°C for 20 s, 55°C for 1 min, and 72°C for 30 min; and a final extension at 72°C for 7 min. Fragments of the BoV VP1/2 region were amplified by nested PCR by AmpliTaq Gold™ 360 Master Mix (Thermo Fisher Scientific Co., Ltd., Massachusetts, USA) in a 50 μL reaction volume. The first-round PCR for 609 bp of BoV VP1/2 region was performed using the following thermal cycling parameters: 95°C for 10 min; 10 cycles of 95°C for 30 s, 58°C for 1 min, and 72°C for 1 min with cycle annealing dropping 0.5 per cycle; 30 cycles of 95°C for 30 s, 54°C for 45 s, and 72°C for 45 s and a final extension at 72°C for 10 min. The second-round PCR for 576 bp of BoV VP1/2 region was performed using the following thermal cycling parameters: 95°C for 10 min; 10 cycles of 95°C for 30 s, 60°C for 1 min, and 72°C for 1 min with cycle annealing dropping by 0.5 per cycle; 30 cycles of 95°C for 30 s, 58°C for 45 s, and 72°C for 45 s and a final extension at 72°C for 10 min. The special primers for HRV and BoV were shown in Supplementary Table 1. The PCR products were purified and sequenced directly (Sanger Biotechnology Co., Ltd., Shanghai, China), and consensus sequences were assembled using BioEdit software, version 7.0.9.

The genotypes of the HRVs and BoVs were identified using the Basic Local Alignment Search Tool^1^ and phylogenetic trees. Phylogenetic trees were constructed using the maximum likelihood method implemented in MEGA software (version 6.06) with 1,000 bootstrap replicates.

### Viruses isolation and treatment by artificial gastric acid or artificial bile/pancreatic juice

Hi-hela cells were selected for HRV isolation. The cytopathic effect was detected by inverted microscope and identified by real-time PCR (Jiangsu Uninovo Biological Technology Co., Ltd., China). Besides, the positive specimens and nucleic acids were incubated for 1, 2, 3, or 4 h with phosphate-buffered saline (PBS), artificial gastric acid or artificial bile/pancreatic juice (Takagi et al., 2003). Artificial gastric acid was adjusted to pH 2 with hydrochloric acid and supplemented with 0.35% pepsin from porcine gastric mucosa and 0.2% NaCl (Cayman Chemical, Kunshan, China). Artificial bile/pancreatic juice contained PBS, 0.2% ox bile and 0.5% pancreatin from hog pancreas (Cayman Chemical, Kunshan, China). Virus RNA copy number (ΔRn) and cycle threshold (Ct) were then measured by real-time PCR (Jiangsu Uninovo Biological Technology Co., Ltd., China). Data are expressed as mean ± SD of three experiment.

### Statistical analysis

The differences in the detection rates of respiratory viruses between 2019 and 2020 were analyzed using the chi-square test and Fisher’s exact test. A two-sided *p*-value of less than 0.05 is considered statistically significant. All analyses were performed using SPSS software version 20.0.

## Results

### The detection of respiratory viruses among diarrhea cases

From 2019 to 2020, 558 fecal specimens from diarrhea cases, including 300 fecal specimens in 2019 and 258 fecal specimens in 2020, were collected in enteric disease clinics, and 16 groups of respiratory viruses were identified, as shown in Table 1. The detection rate for these respiratory viruses was 8.42% (47/558). Forty cases (13.33%, 40/300) were positive in 2019, and 7 cases (2.71%, 7/258) in 2020. The difference in the detection rates in 2019 and 2020 was statistically significant (χ^2^ = 16.648, *p* = 0.00).

**TABLE 1:** The detection of respiratory viruses among diarrhea cases in enteric disease clinics in Beijing from 2019 to 2020.

Virus catalogs	2019 (*n* = 300)	2020 (*n* = 258)	Total	χ^2^	*P*
Human rhinovirus	16 (5.33%)	4 (1.55%)	20 (3.58%)	8.105	0.005
Parainfluenza virus I	6 (2.00%)	0	6 (1.08%)	5.216	0.024
Bocavirus	12 (4.00%)	1 (0.39%)	13 (2.33%)	7.955	0.004
Coronavirus OC43	3 (1.00%)	1 (0.39%)	4 (0.72%)	0.788	0.357
Respiratory syncytial virus A	2 (0.67%)	1 (0.392%)	3 (0.54%)	0.229	0.544
Influenza A&B virus	0	0	0	/	/
Parainfluenza virus II, III, and IV	0	0	0	/	/
Coronavirus 229E, NL63, HKU1	0	0	0	/	/
Metapneumovirus	0	0	0	/	/
SARS-CoV-2	0	0	0	/	/
Respiratory syncytial virus B	0	0	0	/	/
Mixture infection	1 (0.33%)	0	1 (0.18%)	0.862	0.538
Total	40 (13.33%)	7 (2.71%)	47 (8.43%)	16.648	<0.001

**FIGURE 1:**
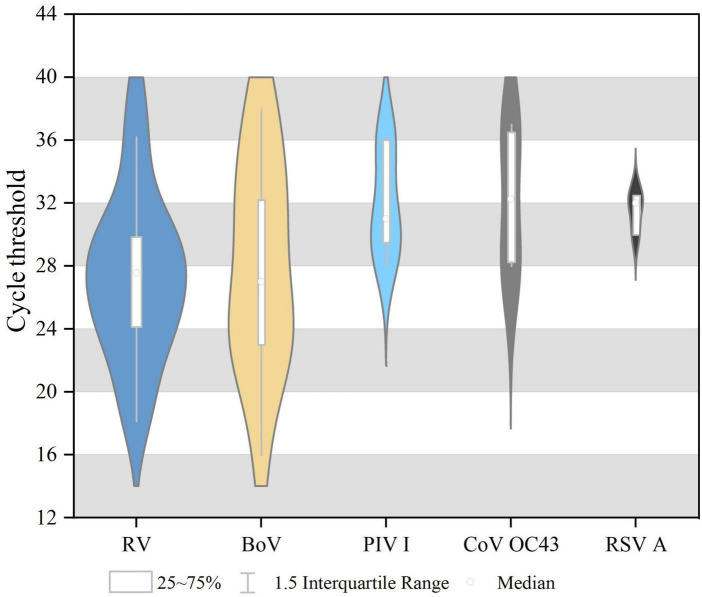
The cycle threshold (CT) values of respiratory viruses among diarrhea patients in acute enteric disease clinics in Beijing from 2019 to 2020.

Single virus infection was found in 46 cases, and one case was a mixture of BoV and CoV OC43. Among 46 cases of single infection, HRV was the most prevalent (3.58%, 20/558), followed by BoV (2.532%, 13/558), PIV I (1.08%, 6/558), CoV OC43 (0.72%, 4/558), and RSV A (0.54%, 3/558). No cases were infected with SARS-CoV-2, IAV, IBV, PIV (II, III, and IV), CoV (229E, NL63, and HKU1), RSVB, or MPV. All these viruses were mostly detected in 2019, and the differences in the detection rates of HRV, PIV I, and BoV between 2019 and 2020 were statistically significant, as shown in Table 1. The Ct values of these positive respiratory viruses among diarrhea patients are shown in Figure 1, and the proportion of Ct blew 30 were 70.00% (14/20) in HRV, 69.23% (9/13) in BoV, 33.33% (2/6) in PIV I, 50.00% (2/4) for CoV OC43, respectively.

### The detection of diarrhea bacteria and parasites among respiratory viruses’ positive cases

Among the 47 respiratory virus-positive cases, an HRV-positive case and a BoV-positive case were detected with EPEC infection, with Ct values of 30 and 34.5, respectively. In addition, no *V. parahaemolyticus, Y. enterocolitica, B. atrophaeus, A. hydrophila, C. jejuni, P. shigelloides, A. hydrophila, C. jejuni, C. coil, C. difficile, V. cholera, V. vulnificus, B. hominis, D. fragilis, E. histolytica, G. lamblia, Cryptosporidium*, and *Escherichia* (EAEC, ETEC, EHEC, and EIEC) positive was detected among these cases.

### Clinical characteristics of patients infected respiratory viruses

The clinical characteristics of the cases infected with a single respiratory virus are shown in Table 2. HRV, BoV, PIV I, COV OC43, and RSV were mostly detected among patients aged 1–3 years, and one patient with HRV infection was 57 years old. The male-to-female ratio in these cases was 30/16. The median duration from onset to visit was 1 [interquartile range (IQR), 1–] for HRV, 2 (IQR, 1–4) for BoV, 2 (IQR, 2–5) for PIV I, 7 (IQR, 2–13) for COV OC43, and 1 (IQR, 1–2) for RSVA. Body temperature was reported in 33 cases infected with single virus, one third of the patients had fever symptoms, and two cases, infected with HRV and BoV, respectively, had high fever with temperature of 39.1 and 40.7°C. These patients mainly had loose feces and watery feces, and only one patient infected with RSV had bloody purulent feces. Maximum number of diarrhea in 1 day were mostly from 3 to 5 times (45.65%, 21/46) and 6 to 10 times (50.00%, 23/46); only 2 cases infected with HRV and BoV, respectively, had diarrhea over 10 times. The patient with mixed infection of BoV and CoV OC43 was a girl of 1 year old who had 6 times of loose feces six times and had a microscopic examination of leukocyte positivity. None of the patients showed symptoms of tenesmus, nausea, dehydration, disturbance of consciousness, systemic poisoning, and shock.

**TABLE 2:** The clinical characteristics of respiratory viruses infections among diarrhea cases in enteric disease clinics in Beijing from 2019 to 2020.

Groups	HRV (*n* = 20)	BoV (*n* = 13)	PIV I (*n* = 6)	COV OC43 (*n* = 4)	RSV (*n* = 3)
**Age (years)**					
0–1	5 (25.00)	3 (23.08)	1 (16.67)	1 (25.00)	0 (0)
1–3	13 (65.00)	9 (69.23)	5 (83.33)	3 (75.00)	3 (100.00)
3–5	1 (5.00)	1 (7.69)	0 (0)	0 (0)	0 (0)
6–55	0 (0)	0 (0)	0 (0)	0 (0)	0 (0)
>55	1 (5.00)	0 (0)	0 (0)	0 (0)	0 (0)
**Gender**					
Male	15 (75.00)	7 (53.84)	3 (50.00)	3 (75.00)	2 (66.67)
Female	5 (25.00)	6 (46.15)	3 (50.00)	1 (25.00)	1 (33.33)
**Time from onset to visit (days)**					
0–1	10 (50.00)	3 (23.08)	0 (0)	1 (25.00)	2 (66.67)
2–7	9 (45.00)	7 (53.84)	5 (83.33)	1 (25.00)	1 (33.33)
>7	2 (5.00)	3 (23.08)	1 (16.67)	2 (50.00)	0 (0)
**Temperature**					
<37.3°C	10 (50.00)	6 (46.15)	4 (66.67)	2 (50.00)	0 (0)
37.3–37.9°C	3 (15.00)	2 (15.38)	0 (0)	0 (0)	0 (0)
38–39.0°C	1 (5.00)	2 (15.38)	1 (16.67)	0 (0)	0 (0)
>39.0°C	1 (5.00)	1 (7.69)	0 (0)	0 (0)	0 (0)
No information	5 (25.00)	2 (15.38)	1 (16.67)	2 (50.00)	3 (100.00)
**Stool properties**					
Loose	15 (75.00)	10 (76.92)	3 (50.00)	1 (25.00)	1 (33.33)
Watery	5 (25.00)	3 (23.07)	3 (50.00)	3 (75.00)	1 (33.33)
Bloody purulent	0 (0)	0 (0)	0 (0)	0 (0)	1 (33.33)
**Maximum times of diarrhea in a day**					
3–5	9 (45.00)	6 (46.15)	4 (66.67)	1 (25.00)	1 (33.33)
6–10	10 (50.00)	6 (46.15)	2 (33.33)	3 (75.00)	2 (66.67)
>10	1 (5.00)	1 (7.69)	0 (0)	0 (0)	0 (0)
Vomiting	0 (0)	0 (0)	2 (33.33)	0 (0)	2 (66.67)
**Microscopic examination of stool**					
Erythrocyte	7 (35.00)	2 (15.38)	2 (33.33)	1 (25.00)	1 (33.33)
Leukocyte	11 (55.00)	9 (69.23)	2 (33.33)	3 (75.00)	2 (66.67)

### Temporal distribution of respiratory viruses

**FIGURE 2:**
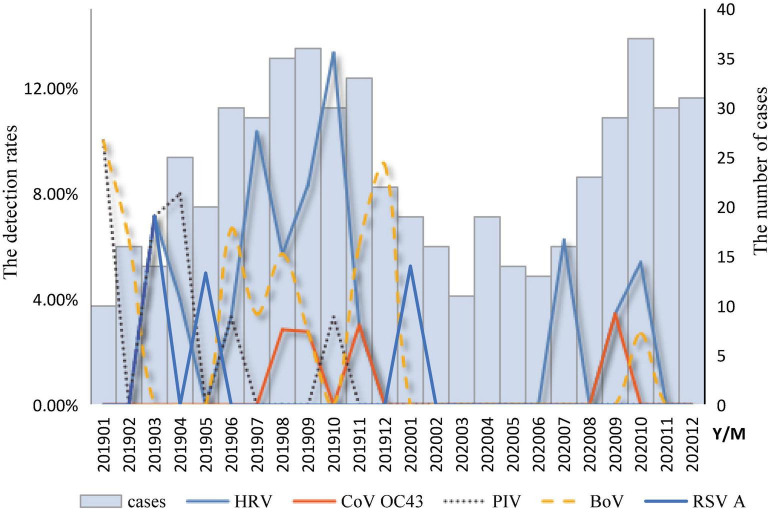
Temporal distribution of respiratory viruses among diarrhea patients in enteric disease clinics in Beijing from 2019 to 2020.

The temporal distribution of cases of infection with a single respiratory virus is shown in Figure 2. The peak months of HRV measurements ranged from July to October. The BoV had two peak months; the first was from June to August, and the second was from November to February of the following year. The peak months of CoV OC43 infection ranged from August to September. The peak months of RSV infection occurred from January to May. The peak months of the PIV ranged from January to June. A mixed infection with BoV and CoV OC43 was detected in August.

### Regional distribution of respiratory viruses

**FIGURE 3:**
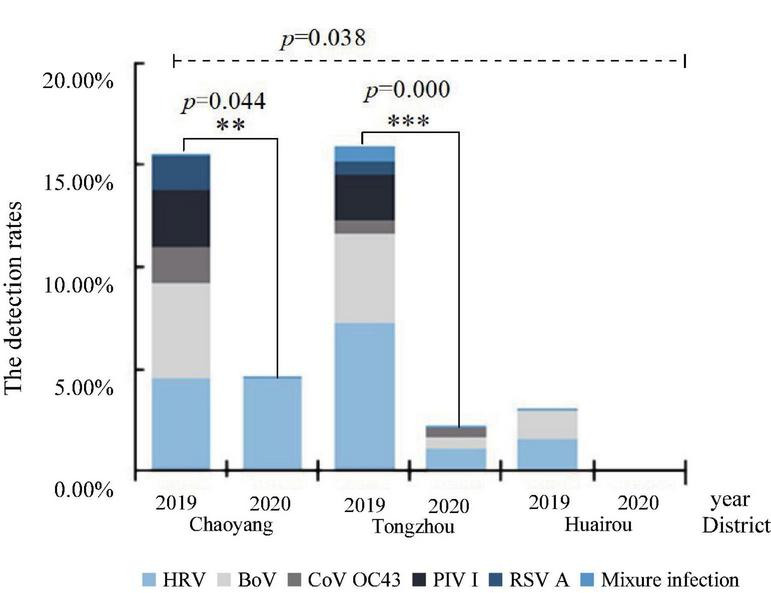
Distribution of respiratory viruses among diarrhea patients in enteric disease clinics in Beijing from 2019 to 2020.

These detection rates of respiratory viruses in Chaoyang, Tongzhou, and Huairou districts were 12.50% (19/152), 8.17% (26/318), and 2.27% (2/88). The detection rates in these districts in 2019 were 16.19% (17/105), 16.92% (22/130), 3.08% (2/65), 4.65% (2/43), 2.22% (4/180), and 0% (0/35) in 2020, respectively. The difference in the detection rates among the three districts in Beijing was statistically significant (*p* = 0.038). HRV, PIV I, BoV, CoV OC43, and RSV A were detected in Chaoyang in the urban area and Tongzhou in the suburb, while only HRV, BoV, and PIV I were detected in Huairou in the outer suburb, as shown in Figure 3. HRV and BoV were the main respiratory viruses in three districts, with the detection rates ranging from 0.83 to 3.83% and 0.83 to 3.80%, respectively.

### Genotyping of HRVs

**FIGURE 4:**
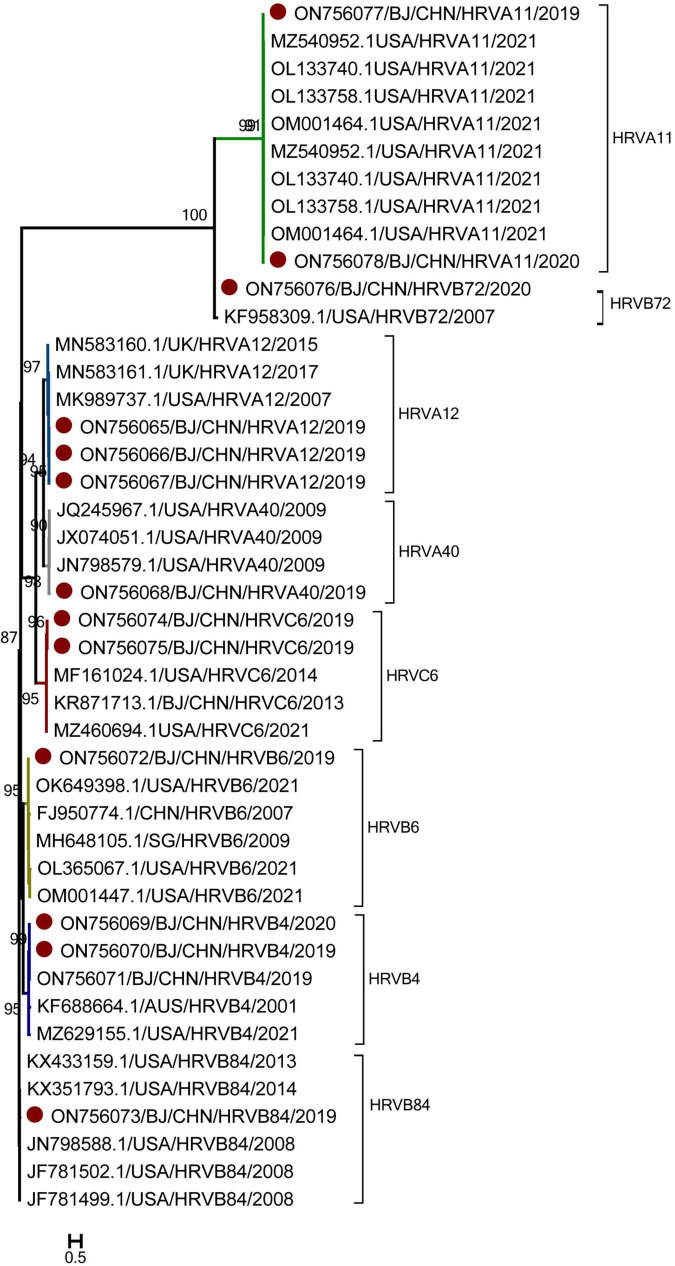
The phylogenetic tree of human rhinovirus (HRV) strains based on VP4 to VP2 gene region. The phylogenetic relationships were estimated by the maximum-likelihood method with GTR + G + I model. HRV strains detected in this study were marked with the symbol:●. Bootstrap values, estimated from 1,000 replicates, are indicated at each node. The scale bar indicates the number of nucleotide substitutions per site.

Genotyping was performed for 14 HRV-positive specimens with Ct values of 30 and for 6 HRV-positive specimens with Ct values ranging from 30 to 40. Thirteen HRV sequences were obtained and compared with the sequences in GenBank. As shown in Figure 4, 13 sequences belonged to three genogroups: HRV-A (6), HRV-B (6), and HRV-C (1). Six HRV-A sequences were identified: HRV-A11 (2), HRV-A12 (3), and HRV-A40 (1). Six HRV-B sequences were identified: HRV-B4 (3), HRV-B6 (1), HRV-B84 (1), and HRV-B72 (1). One sequence of HRV-C belonged to the HRV-C6. All these 13 HRV sequences had the high similar to strains detected in US and shared the nucleotide identities of 97.13 to 97.64% with them.

**FIGURE 5:**
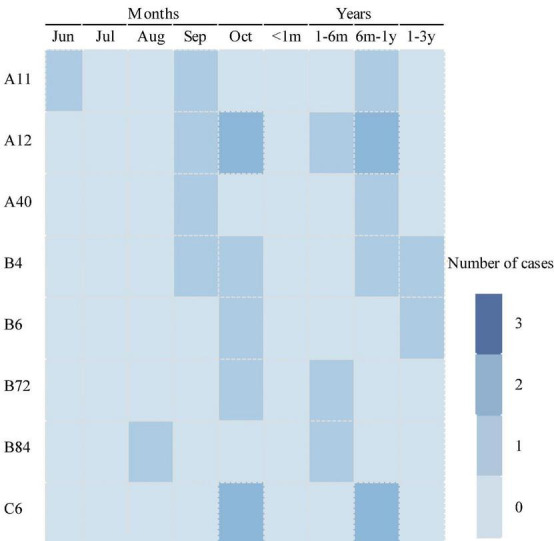
The distribution of human rhinovirus (HRV) genotypes among patients of different ages.

As for the temporal distribution of different genotypes of HRV, all these genotypes were detected in autumn, including HRV-A11, HRV-A40, and HRV-C6 detected in September; HRV-B4, HRV-B6, and HRV-B72 detected in October; and HRV-A12 and HRV-B84 detected both in September and October. Only HRV-A11 was detected in June during the summer. As for the age group shown in Figure 5, except HRV-B6 and HRV-B4, which were detected in cases 1–3 years old, all other genotypes of HRV were detected in cases from 1 month to 1 year of age.

### Genotyping of BoVs

**FIGURE 6:**
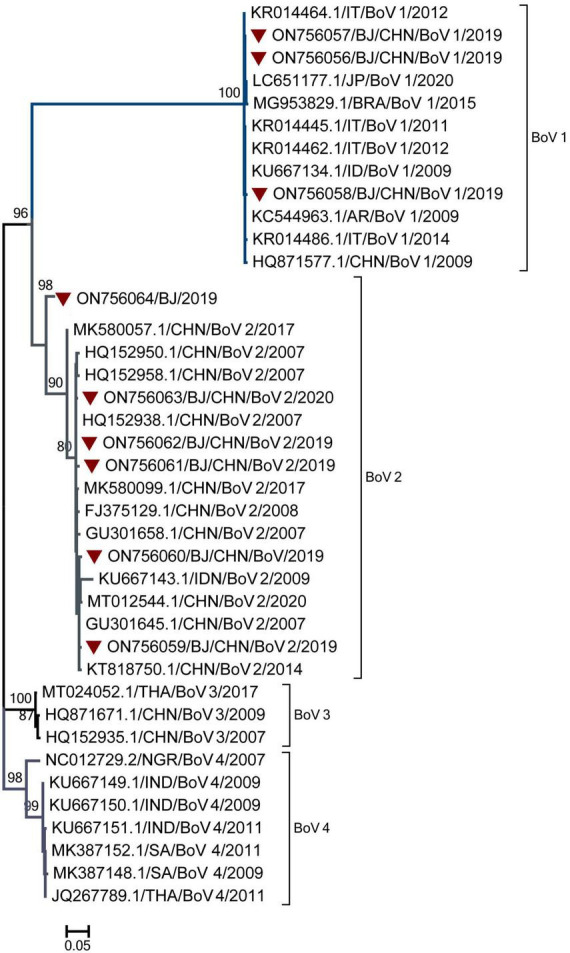
The phylogenetic tree of Bocavirus (BoV) strains based on VP1 to VP2 gene region. The phylogenetic relationships were estimated by the maximum-likelihood method with GTR + G model. Human rhinovirus (HRV) strains detected in this study were marked with the symbol:▼. Bootstrap values, estimated from 1,000 replicates, are indicated at each node. The scale bar indicates the number of nucleotide substitutions per site.

Genotyping was performed for nine BoV-positive specimens with Ct values of 30 and three BoV-positive specimens with Ct values ranging from 30 to 40. Nine BoV sequences were obtained and compared with the sequences in GenBank. As shown in Figure 6, nine sequences belonged to two genogroups: BoV 1 (3) and BoV 2 (6). The three BoV 1 sequences had the high similarity with strains detected in Japan, Brazil, Italy, India, Argentina and shared the nucleotide identities of 99.70 to 99.98% with identities. The five BoV 2 sequences had the high similar to strains detected in Beijing and Guangzhou in China and shared the nucleotide identities of 98.90–99.64% with them. As for the temporal distribution and age group, both BoV 1 and 2 were detected in two peak months from June to August and November to February of the next year and among cases from 1 to 9 years of age.

### The risk of respiratory virus transmission through feces

Among 20 positive HRV specimens from 2019 to 2020, Hi-Hela cells infected with 2 HRV specimens (number: 20,051,039, 19,121,161) showed cytopathic effect and were identified as positive by real-time PCR (Supplementary Figure 1).

**FIGURE 7:**
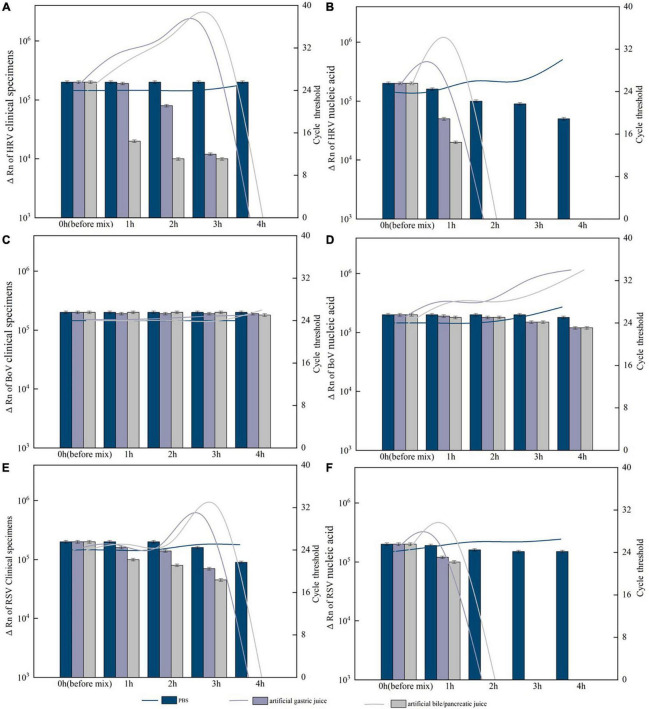
Resistance of the positive specimens and nucleic acids of respiratory viruses to artificial digestive juice (artificial gastric acid and bile/pancreatic juice). **(A)** Resistance of the human rhinovirus (HRV) positive specimens to artificial digestive juice; **(B)** resistance of the HRV nucleic acids to artificial digestive juice; **(C)** resistance of the Bocavirus (BoV) positive specimens to artificial digestive juice; **(D)** resistance of the BoV nucleic acids to artificial digestive juice; **(E)** resistance of the respiratory syncytial virus (RSV) positive specimens to artificial digestive juice; and **(F)** resistance of the RSV nucleic acids to artificial digestive juice. Virus RNA copy number (ΔRn) was shown by bar charts, and cycle threshold (Ct) was shown by line charts.

For HRV, BoV, and RSV with Ct value of 24 and ΔRn ranging from 2.4 to 2.7 × 10^5^, after 4 h of treatment and digestion by artificial gastric acid or artificial bile/pancreatic juice, both clinical specimens and nucleic acid of BoV could be detected and Ct value of the nucleic acid was slightly higher than that of clinical specimens. The clinical specimens of HRV and RSV could not be detected after 4 h of treatment, while their nucleic acids were not detected after 2 h of treatment. For PIV and CoV with Ct value of 28 and ΔRn ranging from 1.0 to 1.2 × 10^5^, both clinical specimens and nucleic acid could not be detected after an hour of treatment. All these clinical specimens and nucleic acid were detected with slightly decreased ΔRn values and slightly increased Ct values after 4 h of treatment by PBS (Figure 7).

## Discussion

Gastrointestinal symptoms occur in COVID-19, and there is the possibility of fecal transmission (Tian et al., 2020). A study in Hubei by Pan et al. (2020) showed that 99 of 204 (48.5%) COVID-19 cases had gastrointestinal symptoms, consistent with 35 (35.35%, 35/99) cases having diarrhea and 6 cases (6.06%, 6/99) manifesting only gastrointestinal symptoms without respiratory symptoms. In addition, previous studies showed that CoV OC43 has a high detection rate, up to 57.00%, in patients with gastrointestinal symptoms; however, only 26.6% to 36.6% in patients with rhinitis, pharyngitis, bronchitis, or bronchiolitis (Freymuth et al., 1997; Vijgen et al., 2005; Openshaw, 2009). The gastrointestinal symptoms caused by CoV may result from direct infection of the gut mucosa, decreased antibacterial defenses, increased mucosal permeability, bacterial translocation, and systemic leakage of endotoxin (Openshaw, 2009). The detection rate of CoV OC43 in diarrhea patients in this study was much lower than the detection rate in the report, and no SARS-COV-2 was detected, which might be due to the interventions for COVID-19 (Openshaw, 2009).

A systematic review showed that the prevalence of influenza viruses in feces among diarrheal cases with influenza ranged from 19.6% (95% *CI*, 9.61 to 32.13) for IVA (H3N2) to 14.42% (95% *CI*, 7.48–23.52) for IVB (Minodier et al., 2015). One prospective observational study found that diarrhea occurred significantly more often in the fecal IAV-positive group (85.71%, 6/7) than negative group (0%, 0/12) among patients infected with IAV (Hirose et al., 2017). In patients infected with IAV and IAB, viral RNA became undetectable in sputum on days 7 and 10 after infection, respectively, but could be detected in feces for a further 2 weeks. IAV virus mRNA and antigens were also detected in the intestinal tissues of one IAV-diagnosed patient (Hirose et al., 2017). No IAV or IBV were detected among diarrheal patients in enteric disease clinics in this study. In Beijing, influenza-like cases were arranged to visit specialized fever clinics, which might have decreased the detection of IAV and IBV among outpatients with diarrhea.

Human rhinovirus has been the leading cause of upper respiratory tract infections (URTIs) since its first isolation in the 1950s, and HRV also causes pneumonia hospitalization in vulnerable people, such as children, the elderly, and those with underlying diseases (Stobart et al., 2017). HRV-associated diseases pose a great socio-economic burden reported in the United States (Fendrick et al., 2003). In Guangzhou, China, the detection rate of HRV was 6.14% (42/655) among children with severe ARTI, and 57.13% (24/42) of cases were children under 2 years old from August 2018 to December 2019 (Li et al., 2021). In this study, the detection rate of HRV in 2019 among diarrhea cases was 5.33% (16/300), and 90% (18/20) of cases were 1 month to 3 years old, which was similar to the results of study in Guangzhou among cases with severe ARTIs (Li et al., 2021). The genogroups of 40 HRV cases identified in Guangzhou, China, were 18 (45%) of HRV-A and 22 (55%) of HRV-C (Li et al., 2021). In Shanghai, China, a total of 29 HRV-A genotypes, 8 HRV-B genotypes, and 22 HRV-C genotypes were detected in the children among ARTI cases from 2019 to 2020, and HRV-A was the most frequently detected species both in the inpatients (51.6%, 163/316) and the outpatients (45.8%, 33/72) caused severe URTIs, followed by HRV-C (27.2%, 86/316 for inpatients; 33.3%, 24/72 for outpatients) and HRV-B (3.5%, 11/316 for inpatients; 11.1%, 8/72 for outpatients), and Among the 59 genotypes identified in these cases, the predominant genotypes were A11, A28, A47, A82, A101, C40, and C43 (Jia et al., 2022). In our study, eight HRV genotypes (A11, A12, A40, B4, B6, B84, B72, and C6) were included among outpatients with diarrhea in Beijing, China, from 2019 to 2020. Among them, two HRV genotypes (A11 and A12) detected in diarrhea cases in our study were also detected in ARTI cases in Shanghai during the same period, and HRV-A11 was the leading genotype in the ARTI cases (Jia et al., 2022). Compared with HRV-positive cases diagnosed with ARTI and diarrhea, both epidemiological characteristics (temporal and population distribution) and the sequences of HRVs were similar, which proved that cases infected with the same pathogen could cause different symptoms of diseases. Therefore, these data provided evidence of the risk of ARTI caused by HRV transmission from diarrhea cases.

From 2005 to 2016, the global BoV prevalence in both children and adults was 5.9% (95% *CI*: 5.7–6.1) for AGE cases and 6.3% (95% *CI*: 6.2–6.4) for ARTI cases (Leitão et al., 2020). In our study, the detection rate of BoV in diarrhea cases was 4.00% (12/300) in 2019, which was very similar to the global BoV prevalence. As for the detection of BoV among fecal and saliva samples from same BoV-positive children in the Amazon diagnosed with AGE and ARTI, the BoV1 was predominantly detected in fecal and saliva samples from AGE children, with 75% (9/12) detection in feces and 100% (11/11) in saliva, the BoV2 was predominant and detected in 71.4% (5/7) of samples from ARTI, and only one sample of the BoV3 was detected in children presenting ARTI (Leitão et al., 2020). In Lanzhou, China, BoV2 was detected in 20.4% of 632 hospitalized children with diarrhea, higher than BoV1 (4.2%) and BoV 3 (0.9%) (Cheng et al., 2011). In this study, both BoV 1 and 2 were detected in patients with diarrhea, and the detection rate of BoV 2 was higher than that of BoV 1. BoV messenger RNAs (mRNA) were only detected in fecal samples and not detected in any saliva samples, including three BoV1 mRNA-positive samples identified from AGE children and one BoV2 mRNA-positive sample identified from an ARTI child (Leitão et al., 2020). Therefore, because BoVs are the common pathogens of AGE and ARTI with similar detection rates and genotypes, the risk of ARTI transmitted from fecal samples of diarrheal cases should be noted.

Compared with that of 2019, the detection rates of HRV, BoV, PIV I, COV OC43, and RSV decreased significantly in 2020 in this study, and no positive cases were detected in the first half of the year except for RSV A. From 2015 to 2021, the detection rates of HRV, BoV, PIV, COV, and RSV among 41,630 ARTIs in Beijing were 3.29, 0.76, 4.54, 2.00, and 2.14% from 2015 to 2019 before COVID-19, and 2.17, 0.41, 1.71, 2.42, and 1.23% from 2020 to 2021 during COVID-19 (Dong et al., 2022). From 2015 to 2019, the monthly detection rates of HRV, RSV, and PIV among 1,579 ARTIs in Huairou districts in Beijing were similar in these years and ranged from 0 to 20.00%, 0 to 15.00%, and 0 to 27.00%, respectively, however, the monthly detection rate in 2020 decreased significantly and only ranged from 0 to 5.00%, 0, and 0 to 5.00%, respectively (Lili et al., 2022). Since the first reports of SARS-CoV-2 infection in China, non-pharmaceutical interventions (NPIs) for COVID-19 (contact tracing, quarantine, mask wearing, social distancing, restrictions on gatherings, etc.) have played an important role in epidemic control (Zhang et al., 2021). NPIs were taken in Beijing since the outbreaks of SARS-CoV-2, and students attended classes online instead of going to school in the first half of the year. It has also been reported that mask wearing is an effective way to reduce HRV transmission. These results suggest that mask wearing and other NPIs might be effective ways to reduce the transmission of HRV, BoV, PIV I, COV OC43, and RSV among patients with diarrhea.

Virus isolation and digestion of clinical specimens and nucleic acid by artificial gastric acid or artificial bile/pancreatic juice were conducted to evaluate the risk of respiratory virus transmission through feces. HRV infectious virions from 2 HRV specimens were isolated successfully in this study. A study examined the intestinal tissues infected with IAV, found transverse colon healing and virus mRNA detected in it (Minodier et al., 2015). Infectious IVA virions were isolated among fecal samples collected during the early stage (day 1 or both days 1 and 2) of infection, and researcher indicated this phenomenon might caused by lower levels of virus at this stage or the lack of standard culture methods (Hirose et al., 2017). In our study, 2 HRV specimens caused cytopathic effect in Hi-Hela cells also with high virus titer that Ct values were 20 and 24, respectively. Besides, in our study, after 4 h of digestion by artificial gastric acid or artificial bile/pancreatic, both clinical specimens and nucleic acid of HRV, RSV PIV, and CoV with low Ct values of 24 and high ΔRn could not be detected and nucleic acid already could not be detected after 2 h of digestion. A study reported a capsule endoscope took 4–5 h to pass through the small intestine (Shibuya et al., 2012). Although clinical specimens and nucleic acid of BoV with DNA genome could not be digested completed by artificial gastric acid or artificial bile/pancreatic juice, a previous study found the BoV mRNAs were only detected in fecal samples and not in any saliva samples (Leitão et al., 2020).

Thus, detection of virus RNA with low Ct values in feces could be a proof for active replication in intestine, but not merely a accidental ingestion. For HRV and BoV with the higher detection rates among these viruses, they *via* cellular receptors called ICAM-1 and NF-kappa B (NF-κB), respectively, which are similar with that of EV and cause similar clinical symptoms as well (Xiao et al., 2001; Du et al., 2015; Liu et al., 2016; Lewis et al., 2018; Shen et al., 2019). For RSV, PIV, and CoV OC43 with the similar detection rates, all of them have the receptors distributed in the intestine. PIV damages inhibitory M2 muscarinic receptors for infection which distribute in gut such as mucosal epithelial cells and cardiac glandular epithelial cells in gastric cardia, small intestine and cecum (Fryer and Jacoby, 1991). RSV interacts with the host fractalkine CX3CR1 receptor to mediates attachment through G protein, expressing on Dendritic cells (DCs) which mediates DCs to access to the intestinal lumen and used to controls adaptive immunity to the entero-invasive pathogens (Niess et al., 2005; Jeong et al., 2015). The major receptor for CoV OC43 is 9-O-acetyl-N-acetylneuraminic acid receptor which can keep CoV OC43 not induce conformational changes in acidic pH conditions. These studies about the receptors of HRV, BoV, RSV, PIV, and CoV also give the supports for the detection, clinical symptoms and active replication in intestine of these virus.

However, this study had several limitations. Because the specimens had been repeatedly frozen and thawed and used for virus detection, we only selected the HRV with enough positive specimens and volume for virus isolation. Therefore, only HRV had the certain evidence for the infectivity of respiratory virus transmission through feces. However, for RNA virus of HRV, RSV, PIV and CoV, we only gave meaningful information that low Ct values of RNA virus could be a proof for active replication in intestine. Beside, as for the specific infectivity of norovirus, rotavirus, sapovirus, astrovirus, and enteric adenovirus for diarrhea, we only included diarrhea cases without these viral infections, which may have caused the higher detection rates of respiratory viruses in this study than in real situations. Furthermore, due to no respiratory tract samples collecting in this study, we did not have opportunity to compare the detection rate of fecal samples with throat/oral swabs among these respiratory viruses which could give us the direction whether the diagnosis using diarrhea could replace or support the conventional methods for detection of the current viruses or could be more useful to reveal the novel biology of these viruses.

In conclusion, respiratory viruses, including HRV, BoV, PIV I, COV OC43, and RSV, were detected in diarrhea cases, which confirmed the risk of respiratory virus transmission among diarrhea cases in enteric disease clinics. No SARS-COV-2 was detected, and a significant reduction in the detection of respiratory viruses in 2020 proved that NPIs were effective against these viruses. Therefore, Our study provides an opportunity to propose a possible relationship between diarrhea and respiratory viruses detected in feces.

## Data availability statement

The datasets presented in this study can be found in online repositories. The names of the repository/repositories and accession number(s) can be found below: https://www.ncbi.nlm.nih.gov/genbank/, ON756056-ON756078.

## Ethics statement

The studies involving human participants were reviewed and approved by 2021 **第**(02)总, Beijing Center for Disease Control and Prevention. Informed consent to participate in this study was provided by the participants’ legal guardian/next of kin.

## Author contributions

QW, ZG, and LS conceived and designed the experiments. LS, HY, and WL performed the experiments. LS and CL analyzed the data. QW, PY, DZ, and LJ contributed to reagents and materials. YT, BL, and YW collected the clinical data. ZG and LS contributed to the writing of the manuscript. All authors reviewed the manuscript.

## Footnotes

1
https://blast.ncbi.nlm.nih.gov/Blast.cgi

## Supplementary material

Supplementary Figure 1 Cytopathic effect in Hi-Hela cells and real-time amplification curve of HRV isolated virions. **(A)** Normal Hi-Hela cells; **(B)** Hi-Hela cells infected with 20051039 strains for 72 h; **(C)** Hi-Hela cells infected with 19121161 strains for 72 h; **(D)** real-time amplification curve of Hi-Hela cells infected with 2 HRV strains.
