# Supplementary material for: Occurrence of respiratory viruses among outpatients with diarrhea in Beijing, China, 2019–2020
Source: Front Microbiol. 2023 Jan 12;13:1073980. doi: 10.3389/fmicb.2022.1073980 (PMC9878210; doi:10.3389/fmicb.2022.1073980)
Supplement: Supplementary file 1 [file Table_1.docx]

**Supplementary Table 1. Primer sequences used for PCR amplification**

| Virus | Primer name | 5’ -3’ sequence | Gene | Length(bp) | Reference |
| --- | --- | --- | --- | --- | --- |
| HRV | HRV-1F | CCGGCCCCTGAATGYGGCTAA | VP4/2 | 688 | Wisdom A et al, 2009 |
|  | HRV-1R | ACATRTTYTSNCCAAANAYDCCCAT |  |  |  |
|  | HRV-2F | ACCRACTACTTTGGGTGTCCGTG | VP4/2 | 563 |  |
|  | HRV-2R | TCWGGHARYTTCCAMCACCANCC |  |  |  |
| BoV | AK-VP-F1 | CGCCGTGGCTCCTGCTCT | VP1/2 | 609 | Kapoor Aet al, 2010 |
|  | AK-VP-R1 | TGTTCGCCATCACAAAAGATGTG |  |  |  |
|  | AK-VP-F2 | GGCTCCTGCTCTAGGAAATAAAGAG | VP1/2 | 576 |  |
|  | AK-VP-R2 | CCTGCTGTTAGGTCGTTGTTGTATGT |  |  |  |
