# Supplementary figures and images for: Occurrence of respiratory viruses among outpatients with diarrhea in Beijing, China, 2019–2020
Source: Front Microbiol. 2023 Jan 12;13:1073980. doi: 10.3389/fmicb.2022.1073980 (PMC9878210; doi:10.3389/fmicb.2022.1073980)

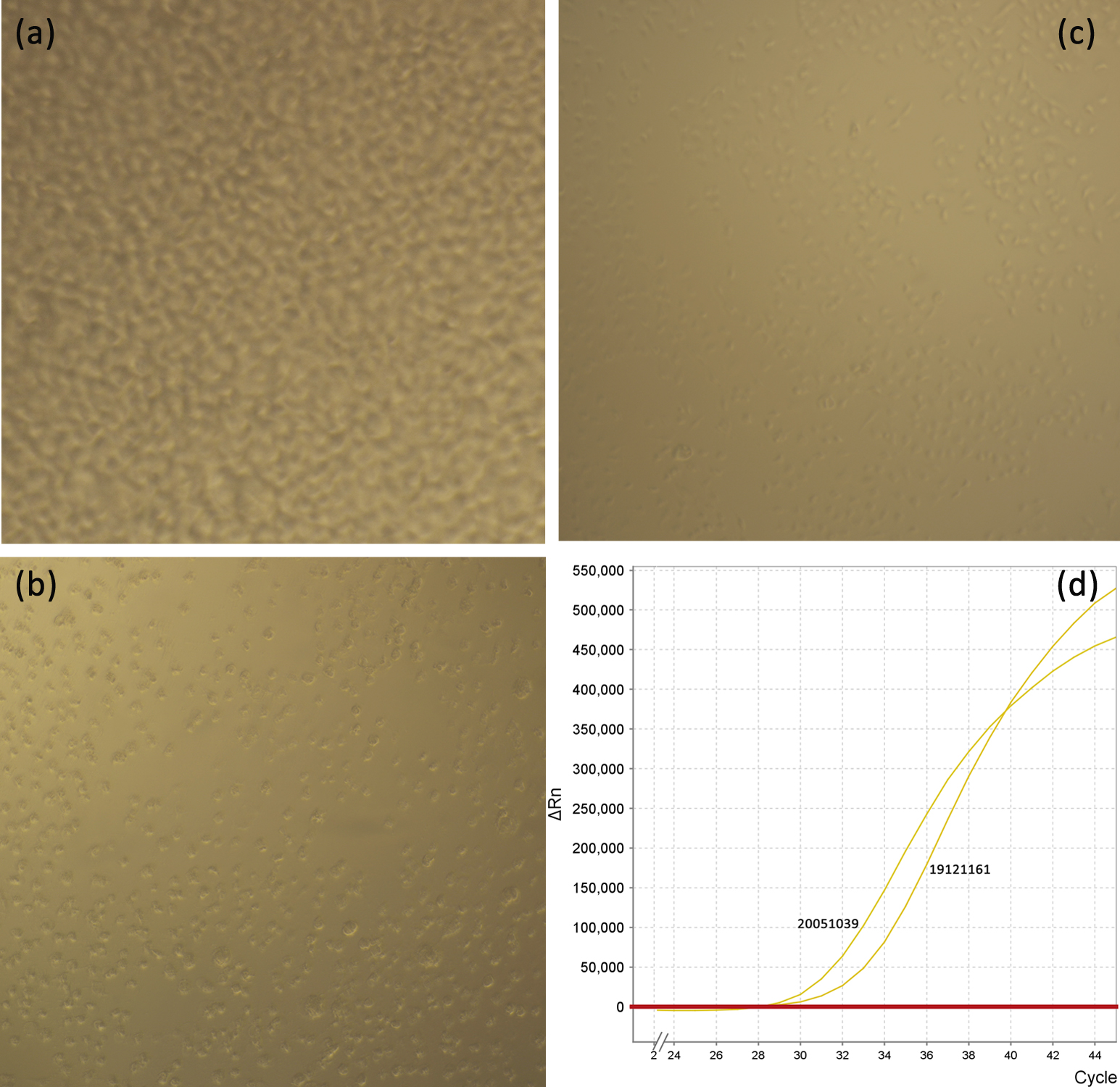

Supplement: Supplementary Figure 1 — Cytopathic effect in Hi-Hela cells and real-time amplification curve of HRV isolated virions. (A) Normal Hi-Hela cells; (B) Hi-Hela cells infected with 20051039 strains for 72 h; (C) Hi-Hela cells infected with 19121161 strains for 72 h; (D) real-time amplification curve of Hi-Hela cells infected with 2 HRV strains. [file Image_1.JPEG]
